# Supplementary material for: High-Accuracy Neural Network Interatomic Potential for Silicon Nitride
Source: Nanomaterials (Basel). 2023 Apr 13;13(8):1352. doi: 10.3390/nano13081352 (PMC10145480; doi:10.3390/nano13081352)
Supplement: Supplementary file 1 [file nanomaterials-13-01352-s001.zip › nanomaterials-2311742-supplementary.pdf]

SUPPLEMENTARY MATERIAL

High accuracy neural network interatomic potential for silicon nitride

Hui Xu[1], Zeyuan Li[2], Zhaofu Zhang[1], Sheng Liu[1], Shengnan Shen[1,*], Yuzheng Guo[3,*],

1 The Institute of Technological and Science, Wuhan University, Wuhan, 430072, China

2 School of Power and Mechanical Engineering, Wuhan University, Wuhan, 430072, China

3 School of Electrical and Automation, Wuhan University, Wuhan, 430072, China

* Correspondence: yguo@whu.edu.cn (Y.G.); shen_shengnan@whu.edu.cn (S.S.)

1. Detailed parameters for Si_3N_4 simulations

The model for the heating process was built based on the structure in the AIMD calculation. There are 10752 atoms in the model, and the cube size in the x, y, and z directions is 51.3 Å, 47.0 Å, and 46.8 Å, respectively. The boundary conditions of the Si_3N_4 box are periodic in three dimensions. The density of Si_3N_4 is about 3.2 g/cm^3 [1-4]. The energy minimization of the system was performed before simulations with the conjugate gradient algorithm. The heating process was simulated in the NVT ensemble for 100 ps with 1 fs timestep, from 1000 K to 3000 K. The Nose–Hoover thermostat was used to control the temperature. The damping parameter for the thermostat is 0.1.

2. Heating process of SiN_x

The RDFs of SiN_x with different temperatures and compositions are shown in Figure S1. The RDFs at given composition during the heating process exhibit that the first peaks of $g(r)$ decrease with increasing temperature. No matter what temperature is applied, the location of the first peaks is around 1.7 Å, indicating that temperature only affects the height of the peak value and will not affect the position. Regarding SiNx, the variation of RDFs in different temperatures decreases with the decrease of x. On the contrary, the valleys of $g(r)$ are proportional to the temperature.

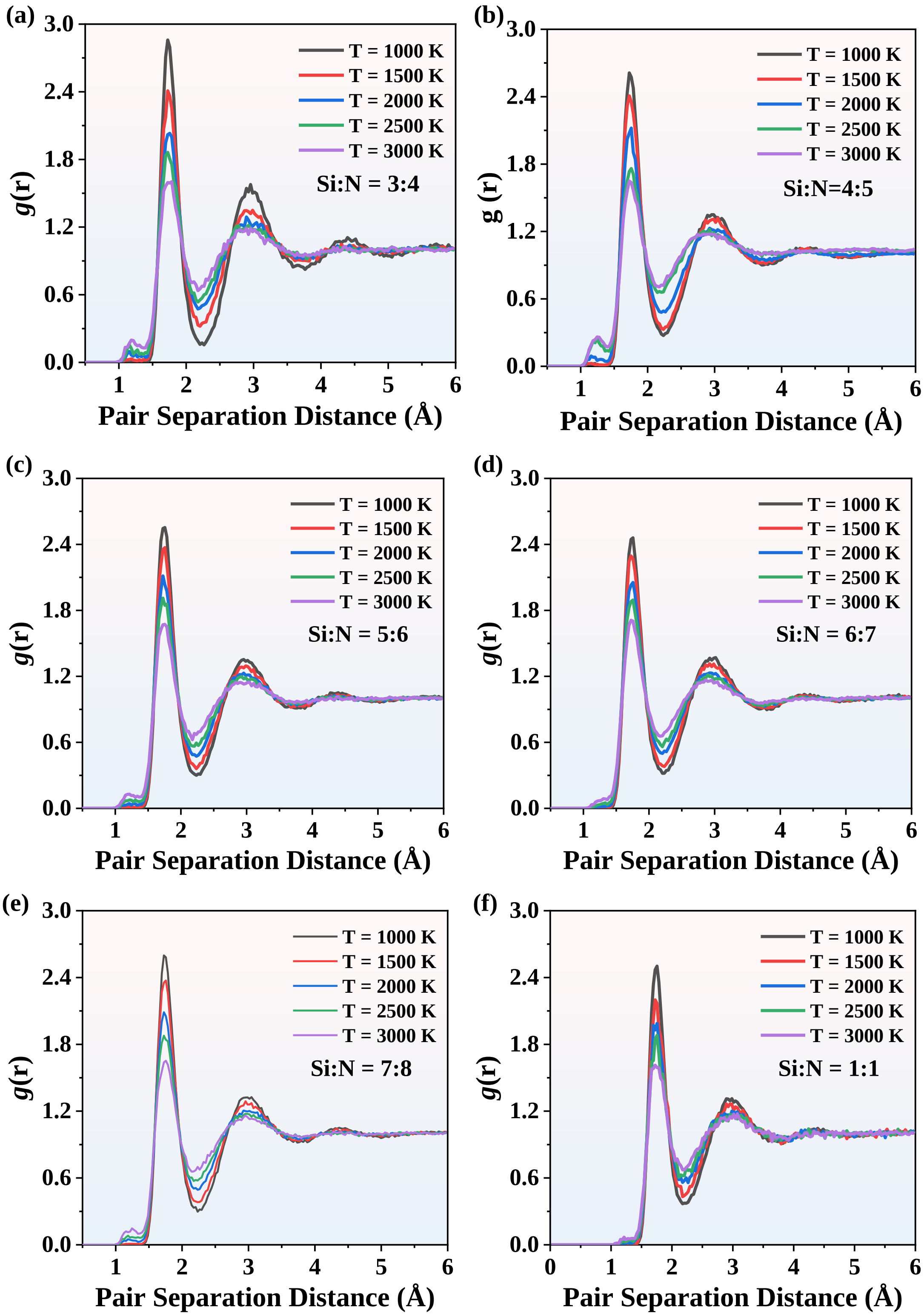

Figure S1. The RDFs of SiN_x with different compositions in the heating process. In (a-f), five temperature stages from 1000 K to 3000 K are chosen, and the first peaks of g(r) decrease with the increasing temperature.

3. Tensile tests of SiN_x

With the analysis of RDFs, CNs, and BADs, the potential we trained is confirmed applicable

to the SiN_x model with the ratio between 3:4 and 1:1. The model selected in the tensile experiment was a cuboid structure, which was uniaxially stretched along the y-axis direction. There were 109760 atoms in the tensile model, which consisted of 47040 Si atoms and 62720 N atoms. The lengths of the x, y, and z directions were 93.004 Å, 200.393 Å, and 82.287 Å, respectively. The three directions were periodic boundary conditions. The system should be relaxed to reach equilibrium before stretching with 1 fs of step size. The SiN_x model was calculated under the NVT ensemble with 100 ps. The next 50 ps simulations were carried out in the NPT ensemble at zero pressure with the Nose–Hoover thermostat and barostat. The damping parameters for the thermostat and barostat were 0.1 ps and 0.5 ps, respectively. When the energy and volume of the system reached the most stable state, the tensile simulations were carried out for 350 ps in the NVT ensemble along the Y direction. To exclude the velocity and force at the boundary end, the 10 layers of atoms at both sides of the Y axis were fixed before stretching. The stretching command was 'fix deform,' and the rate value was 10^{-9}/s.

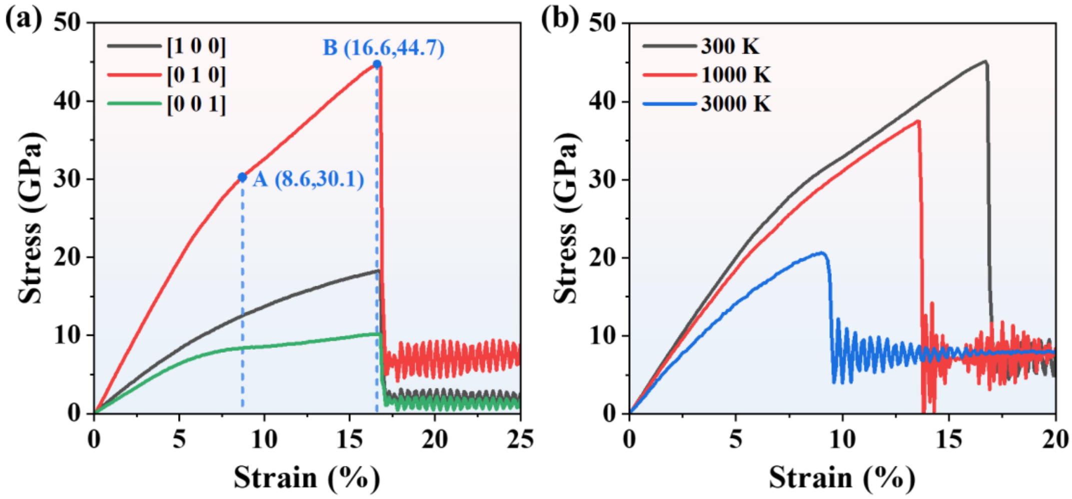

Figure S2. Amorphous Si_3N_4 tensile simulations. (a) The tensile simulations at 300 K. The three curves are stress distributions in different orientations. (b) Amorphous Si_3N_4 tensile simulations comparisons in the Y direction with 300 K, 1000 K, and 2000 K temperatures.

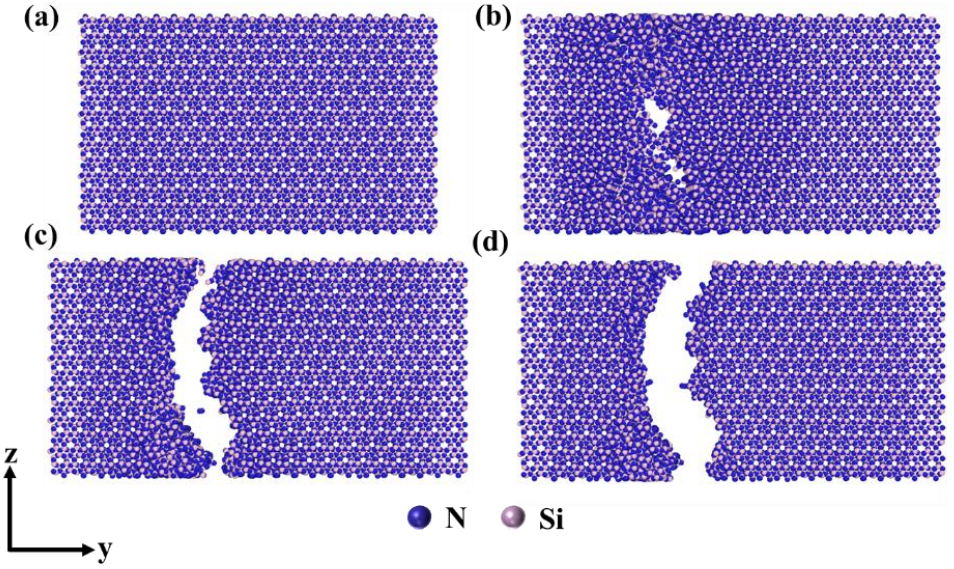

Figure S3. The whole process of fracture during tensile simulations. The (a) initial state, (b) beginning state of fracture, (c) intermediate state, and (d) final state of Si_3N_4 model.

Figure S2 and Figure S3 show the results of tensile simulations. There are four different states during the tensile process in Figure S3, which can be found in the strain–stress curve in Figure S2 (a). Figure S3 (a) exhibits the initial state of the model, which corresponds to point O (O is the origin point) in Figure S2 (a). Figure S3 (b) shows the beginning state of fracture, corresponding to point A in Figure S2 (a). Figure S3 (c) and (d) show the fractured state of the Si_3N_4 model, which represent the state after point B in Figure S2 (a).

The three strain–stress curves in different orientations during the tensile process are displayed in Figure S2 (a). The stress in the [0 1 0] direction is the largest. For the red curve, the stress is 0 GPa at the beginning of the tensile test in Figure S2 (a). With the increase in strain, the stress value increases linearly at first. When strain is 8.6% at point A, the slope decreases. When strain is 16.6% at point B, the stress sharply declines. Then, the whole tensile simulations finished at point B. The initial linear behavior corresponds to an elastic deformation in the tensile process. Elastic modulus is a physical quantity that measures the mechanical properties of materials. In Figure S2 (a), the slope of OA is the elastic modulus E, which is 349.7 GPa. The sharp decline at point B means the model has reached a fractured state. Then, the stress reaches a steady state, which is unchanged with the strain.

To observe the temperature effect on the elastic modulus of Si_3N_4, tensile simulations were calculated at 1000 K and 2000 K. The models used for simulations are the same as that used at 300 K. The variation of strain–stress curves at three temperatures is shown in Figure S2 (b).

It can be concluded that the slope of the strain–stress curve decreases with the temperature increasing. On the other hand, the fracture occurred earlier at high temperatures. The stress declines when strain is 0.166, 0.136, and 0.091 at 300 K, 1000 K, and 2000 K, respectively. The three curves share the same profile after the fracture, all at equilibrium.

Table S1 shows the specific parameters of SiN_x tensile models with different compositions. Parameter x represents the ratio of the number of nitrogen atoms to the number of silicon atoms.

Table S1 The specific simulation parameters in SiN_x tensile simulations.

**Si: N**	**x (Å)**	**y (Å)**	**z (Å)**	**Atoms**
3:4	71.61	143.2	69.3	63504
4:5	75.6	151.2	75.6	74088
5:6	75.6	151.2	75.6	75460
6:7	75.6	151.2	75.6	80262
7:8	75.6	151.2	75.6	72030
1:1	75.6	151.2	75.6	74088

References

1. Bonati L., M. Parrinello, Silicon liquid structure and crystal nucleation from ab-initio deep metadynamics, *Phys. Rev. Lett.* **2018**, *121*, 265701. http://doi.org/10.1103/PhysRevLett.121.265701.
2. Gismatulin A. A., G. N. Kamaev, V. N. Kruchinin, V. A. Gritsenko, O. M. Orlov, A. Chin, Charge transport mechanism in the forming-free memristor based on silicon nitride, *Sci. Rep.* **2021**, *11*, http://doi.org/10.1038/s41598-021-82159-7.
3. Rettore R. P., M. A. M. Brito, Mechanical properties of silicon-nitride bonded silicon-carbide refractory and its relation microstructure. In *Silicon Nitride 93*, M. J. Hoffmann, P. F. BecherG. Petzow, Eds. Trans Tech Publications: Clausthal Zellerfe, 1994; Vol. 89-91, pp. 553-557.
4. Wiederhorn S. M., G. D. Quinn, R. Krause, High-temperature structural reliability of silicon-nitride. In *Silicon Nitride 93*, M. J. Hoffmann, P. F. BecherG. Petzow, Eds. Trans Tech Publications: Clausthal Zellerfe, 1994; Vol. 89-91, pp. 575-580.
